# Extraction and Characterization of Starches from Non-Conventional Sources: Turmeric (*Curcuma longa*) and Mangarito (*Xanthosoma sagittifolium*)

**DOI:** 10.3390/polym17233157

**Published:** 2025-11-27

**Authors:** Gislaine Ferreira Nogueira, Carlos Wanderlei Piler de Carvalho, José Ignacio Velasco, Farayde Matta Fakhouri

**Affiliations:** 1 Department of Biomedical and Health Sciences, Minas Gerais State University, Passos 37900-106, MG, Brazil; gislaine.nogueira@uemg.br; 2 Embrapa Food Technology, Americas Av., 29501, Rio de Janeiro 23020-470, RJ, Brazil; carlos.piler@embrapa.br; 3 Poly2 Group, Department of Materials Science and Engineering, Universitat Politècnica de Catalunya, Carrer Colom 11, E-08222 Terrassa, Spain; jose.ignacio.velasco@upc.edu

**Keywords:** non-conventional starches, amylose content, physicochemical characterization

## Abstract

The characterization of alternative starch sources is crucial for industrial applications. This study evaluated starches from turmeric (*Curcuma longa* L.) and mangarito (*Xanthosoma riedelianum*), considering extraction yield, proximate composition, amylose content, morphology, hydration properties, viscoamylographic behavior, and crystalline and thermal characteristics. Mangarito starch showed a higher yield (11.6%) than turmeric starch (5.6%). Turmeric granules were heterogeneous (triangular, ellipsoidal, oval), while mangarito granules were predominantly rounded. Turmeric starch exhibited higher amylose content (55.1%) compared to mangarito starch (25.9%). Hydration and viscoamylographic analyses indicated that turmeric starch had higher solubility (2.36%) and water absorption (2.88 g/g), higher peak viscosity (3147.5 cP), lower breakdown (83.5 cP), and greater retrogradation tendency (9806 cP). In contrast, mangarito starch demonstrated enhanced thermal stability (breakdown 1824 cP; final viscosity 4763.5 cP). X-ray diffraction revealed a semicrystalline A/B-type pattern for turmeric starch and a predominantly A-type crystalline structure for mangarito starch. DSC indicated glass transition temperatures of 114.7 °C (turmeric) and 120.1 °C (mangarito), while TGA confirmed greater thermal stability for mangarito starch, with a narrower decomposition range and higher residual mass. These results suggest that turmeric starch, due to its high amylose content, is suitable for rapid gelatinization and firm gel formation, whereas mangarito starch is more appropriate for applications requiring superior thermal stability and structural integrity.

## 1. Introduction

The growing demand for ingredients with specific functionalities in the food industry has driven the exploration of new starch sources, aiming to meet technological requirements not met by conventional starches [1].

Starch is widely used as a thickener, stabilizer, texturizing agent, and gel former and is essential in processed, frozen, and ready-to-eat products [2]. Although starch extracted from corn, cassava, rice, and potatoes dominates the market due to its availability and affordable cost, these materials have limitations in applications that require greater thermal stability, resistance to retrogradation, or rapid gelatinization. Thus, conventional starches often require chemical or physical modifications to meet industrial requirements, which can increase costs and reduce the natural appeal of products [2].

In this context, roots and rhizomes of native or underutilized plants, such as turmeric (*Curcuma longa* L.) [3] and the mangarito (*Xanthosoma riedelianum*) [4], have been investigated as alternative sources of starch. Turmeric (*Curcuma longa* L.) belongs to the Zingiberacea family and is a rhizome characterized by a high concentration of curcuminoids, compounds that give it a vibrant yellow to orange color and are widely used as dyes, especially in the food industry [3,5]. In 2020, global turmeric production was estimated at around 1.1 million metric tons, and forecasts point to an average annual growth of 16.1% through 2028 [5,6]. Studies indicate that turmeric has starch granules with heterogeneous morphology (flat triangular shape), amylose content of 22%, and high peak viscosity (740 RVU), in addition to good thermal stability of the paste and a tendency to retrogradation, characteristics that favor its use in products that require firm gels and stable consistency after cooling [3,7].

Mangarito (*Xanthosoma sagittifolium*), in turn, belongs to the Araceae family and is an unconventional food plant whose rhizome, rich in starch, has been valued as an alternative to diversify the food base in socioeconomically vulnerable territories [8]. Mangarito production is still limited, but studies on its cultivation are on the rise, bringing news about techniques to increase productivity and improve the commercial quality of the product [8]. The mangarito starch granules are rounded and 12.5 μm in diameter. The starch demonstrated satisfactory thermal stability even under mechanical agitation, in addition to showing a high propensity for retrogradation [9].

The botanical origin of starch plays a decisive role in determining its physicochemical properties, including the relative proportions of amylose and amylopectin, morphological characteristics, degree of crystallinity, and thermal and nutritional properties [1].

Due to the increasing demand for starches in various sectors—such as food formulation, cosmetics, and pharmaceuticals—there is growing interest in exploring alternative sources capable of meeting commercial requirements [10]. In this context, comparative studies of starches from different plant origins, particularly from rhizomatous and underutilized species such as turmeric (*Curcuma longa* L.) and mangarito (*Xanthosoma sagittifolium*), are essential to identify distinctive functional and technological characteristics.

These species share similar ecological requirements and cultivation conditions, making them suitable for comparative evaluation. Furthermore, as native or underexploited crops in Brazil, their valorization contributes to the diversification of starch sources and the sustainable use of local biodiversity. Understanding the morphological, gelatinization, and thermal properties of these starches can provide valuable insights into their potential domestic and industrial applications [10]. Therefore, this study aimed to extract and characterize starches from turmeric (*Curcuma longa* L.) and mangarito (*Xanthosoma sagittifolium*), comparing their yield, proximate composition, morphological features, hydration properties, and gelatinization behavior in order to identify their potential as alternative and functional starch sources.

## 2. Materials and Methods

### 2.1. Location Where the Experiment Was Conducted and Raw Materials

The experiment was conducted with turmeric (*Curcuma longa* L.) and mangarito (*Xanthosoma sagittifolium* (L.) Schott) plants, cultivated in the area of the Didactic-Scientific Laboratory for Production and Post-harvest of Medicinal Plants (HPM) of the Faculty of Agricultural Sciences of the Federal University of Grande Dourados (UFGD), located in the municipality of Dourados, State of Mato Grosso do Sul, Brazil. The experimental area is located at the geographical coordinates 22°11′47″ S and 54°56′06″ W, at an altitude of 430 m. Turmeric (*Curcuma longa* L.) and mangarito (*Xanthosoma mafaffa* Schott) plants were obtained from the same experimental area and cultivated under conventional management practices. The sampling procedure consisted of the random collection of representative plants from each species, totaling approximately 3 kg of fresh rhizomes per species. Rhizomes were manually harvested at the physiological maturity stage between April and May 2017.

After harvesting, the rhizomes were selected for uniformity in size and the absence of mechanical damage or signs of deterioration. Subsequently, the samples were washed with running water to remove adhering soil particles and air-dried at room temperature until starch extraction, which was performed within a maximum of 48 h after collection.

### 2.2. Extraction of Turmeric and Mangarito Starches

The starches from turmeric and mangarito were extracted following the methodology proposed by Cruz and El-Dash [11] and Nogueira eta al. [12], with modifications. Initially, the rhizomes were selected, peeled and cleaned with distilled water. They were then cut into slices and immersed for 15 min in a 0.03% (m/m) potassium metabisulfite solution to prevent oxidation. After this treatment, the rhizomes were ground in a stainles steel industrial blender (Spolu, Itajobi, SP, Brazil), using a 1:2 (m/m) rhizome to distilled water ratio, for a period of 5 min, until a homogeneous paste was formed.

The paste obtained was then filtered through double cotton cloth, and the retained material was subjected to three consecutive washes with deionized water to remove the fibers and completely recover the starch. The supernatant was left to stand for approximately 12 h, allowing the natural sedimentation of the starch, which was then manually separated from the liquid phase. The starchy sediment was dried in an oven with air circulation at 40 °C for 5 h. After drying, the starch was macerated in a mortar and stored in a polyethylene plastic bag, remaining at room temperature until its characterization.

### 2.3. Characterization of Starch

#### 2.3.1. Yield of Starch Extraction

To determine the extraction yield, the rhizomes were weighed after selection, as well as the starch obtained after drying [12]. The yield was calculated using Equation (1):

(1) $$\mathrm{Extraction}\;\mathrm{yield}\;(\%)\;=\;\left(\frac{Mds}{Mrp}\right)\times 100$$

where Mds: mass of the dried starch (g); Mrp: mass of the raw plant material (g).

#### 2.3.2. Proximal Composition and Amylose Content

The proximate composition of starches from turmeric and mangarito was analyzed following the official methods described by Association of Official Analytical Chemists (AOAC) [13]. Moisture content was determined gravimetrically by drying the samples in a convection oven at 105 °C for 24 h. Lipid content was measured gravimetrically after extraction with petroleum ether using a Soxhlet apparatus. Protein content was determined by the Kjeldahl method, while ash content was estimated by incineration in a muffle furnace. Total carbohydrate content was calculated by difference, subtracting the sum of moisture, protein, lipid, and ash contents from 100%.

Amylose content was determined by a colorimetric method, as described by Zavareze et al. [14], using a 2% iodine solution, with sample readings performed on a spectrophotometer (Marconi, Jenway 7310, St Albans, UK) at 610 nm.

#### 2.3.3. Water Absorption Index and Solubility Index

The water absorption and solubility indices were determined according to the Schoch [15] methodology. Suspensions containing 0.2 g (dry basis) of starch in 18 g of distilled water were placed in centrifuge tubes and incubated at controlled temperatures from 30 to 90 °C for 30 min in a Dubnoff metabolic bath (model SL157, Solab, Piracicaba, SP, Brazil), with gentle agitation at 150 rpm every 5 min. After incubation, the volume of each sample was adjusted to 20 g with distilled water, homogenized, and centrifuged at 4010 rpm for 15 min. The supernatant was dried in an oven at 105 °C until constant weight, while the remaining gel in the tube was considered as wet sediment and weighed.

The water absorption index (WAI) and water solubility index (WSI) were calculated using Equations (2) and (3), respectively, described by Nogueira et al. [12].

(2) $$WAI\;\left(g\cdot g^{-1}\right)=\frac{Wg}{W-WS}$$

(3) $$WSI\;\left(\%\right)=\frac{Ws}{W}\times 100$$

where ‘Wg’ was weight of sediment (g), ‘W’ was weight of dry solids in original sample (g) and ‘Ws’ was weight of dissolved solids in supernatant (g).

#### 2.3.4. Viscoamylographic Properties

The paste viscosity was assessed according to method AACC 22-10 [16]. The pasting properties of the starch samples were determined using a Rapid Visco Analyser (RVA). For the analysis, 3 g of starch were dispersed in 25 mL of distilled water. The equipment was operated at a rotation speed of 960 rpm during the first 10 min, and then adjusted to 160 rpm for the remainder of the test. The suspension was heated from 25 to 95 °C at an approximate heating rate of 14 °C·min^−1^, followed by cooling to 26.4 °C. During the test, viscosity profiles were recorded as a function of temperature and time, allowing the determination of characteristic pasting parameters, including peak viscosity, trough viscosity, breakdown, final viscosity, and setback. Viscosity was expressed in the cP unit.

#### 2.3.5. Scanning Electron Microscopy (SEM)

The surface morphology of the starch granules was examined by scanning electron microscopy (SEM) using a Jeol microscope (model JMS-T330, Tokyo, Japan) operated at 10 kV.

#### 2.3.6. X-Ray Diffraction (XRD)

Diffractograms were acquired using a Philips X’Pert X-ray diffractometer (Amsterdam, The Netherlands) under the following conditions: voltage of 40 kV and current of 40 mA. Scanning was performed over a 2θ range from 5° to 30°, with a step size of 0.1° and a scan rate of 1°/min, equipped with a graphite secondary beam monochromator. Crystal size variation was determined using the PC-APD Diffractometry Software. Samples were stored at 25 °C and 50% relative humidity.

#### 2.3.7. Differential Scanning Calorimetry (DSC)

The thermal properties of the starch samples were determined using a Differential Scanning Calorimeter (DSC, model TA 2010, TA Instruments, New Castle, DE, USA) equipped with a liquid nitrogen cooling system. The samples were prepared and preconditioned at 25 °C and 50% relative humidity prior to analysis. Measurements were performed under an inert atmosphere of ultra-dry, chromatographic-grade nitrogen gas, with both purge and carrier flow rates set at 50 mL min^−1^. The analyses were carried out with an excess of water, starting at 25 °C, and the samples were subsequently heated at a rate of 10 °C min^−1^ up to a final temperature of 160 °C. Atmospheric air was used as the reference material.

#### 2.3.8. Thermogravimetric Analysis (TGA)

Thermogravimetric analysis of the starch samples was carried out using a TGA-50 M instrument (Shimadzu, Kyoto, Japan). Approximately 10 mg of each sample were placed in a platinum crucible and heated from 25 to 600 °C at a constant heating rate of 10 °C/min. The analyses were conducted under a nitrogen atmosphere maintained at a flow rate of 30 mL/min to ensure an inert environment during thermal degradation. The TGA curves were used to determine mass loss as a function of temperature, while the corresponding derivative thermogravimetric (DTG) curves were evaluated to identify degradation stages and compare the temperatures associated with the maximum decomposition rates.

### 2.4. Statistical Analysis

All analyses were carried out in triplicate, except for amylose content, which was analyzed in quadruplicate. The results were expressed as mean ± standard deviation. The data were subjected to statistical comparison using Student’s *t*-test at a 5% significance level (*p* < 0.05) to evaluate differences between the starches from turmeric (*Curcuma longa* L.) and mangarito (*Xanthosoma riedelianum*). Statistical analyses were performed using the Statistica 9.0 software (Stat-Soft, Tulsa, OK, USA).

## 3. Results and Discussion

### 3.1. Yield of Starch Extraction

The yield of starch extracted from turmeric rhizome (*Curcuma longa* L.) was 5.6%, a relatively low value compared to other rhizomes, such as arrowroot (*Maranta arundinacea* L.), which showed a yield of up to 11.81% [17]. In contrast, studies using mango (*Mangifera indica*) seeds as raw material reported significantly higher yields, ranging from 44.49% to 62.11% [18] and from 52% to 65% [19], depending on the extraction conditions.

This low extraction efficiency may be related to the high concentration of non-starch compounds, such as essential oils, curcuminoids and fibers, which are abundant in this rhizome and may interfere with the starch extraction and purification process [3]. Furthermore, factors such as the extraction technique used, the rhizome maturation time and the environmental conditions during cultivation can also negatively impact yield. Maniglia et al. [3] investigated the extraction of starch from the residue of turmeric dye extraction, using maceration in acidic medium, in water and in alkaline medium. The yields obtained varied between 24% and 31%. The low efficiency was attributed to the retention of starch granules in the bran structure (47 to 48% dry basis), composed of fibers, proteins and lipids. Similarly, Panchal and Singh [20] analyzed the effects of physical and chemical methods on the extraction of starch from chayote tubers. The physical method, based on extraction with water, presented the highest yield (21.62%), being comparable to those obtained by chemical methods using NaOH and Na_2_S_2_O_5_.

On the other hand, mangarito starch (*Xanthosoma riedelianum*) showed a significantly higher yield of 11.6% compared to saffron starch. This value was also higher than that reported by Martins et al. [4], who found an extraction yield of 7.89% for starch from *X. riedelianum*. This variation in the starch content present in the rhizomes can be attributed to the physiological composition of mangarito, which has a high concentration of starch stored as an energy reserve, especially when compared to saffron. In addition, it is important to emphasize that the yield can vary depending on several factors, such as the harvest time, the degree of maturity of the rhizomes [21] and the edaphoclimatic conditions, including the type of soil and water availability. According to the study by Zou et al. [22], the starch yields of Chinese yam in the expansion and dormant stages were 63.4% and 61.2%, respectively, evidencing the influence of the tuber growth stages.

The comparison between the two materials shows that mangarito is more viable as an alternative source of starch, especially in regions where this plant is traditionally cultivated. Although turmeric has recognized functional and nutraceutical value due to the presence of curcumin, its low starch productivity limits its use as the main raw material for industrial applications based on this biopolymer. In general, the results indicate that, from a quantitative point of view, mangarito has greater potential as a source of starch, while turmeric may be more suitable for the extraction of bioactive compounds, with starch being a byproduct of less expressiveness.

### 3.2. Proximal Composition and Amylose Content

Table 1 shows the centesimal composition of starches extracted from turmeric (*Curcuma longa* L.) and mangarito (*Xanthosoma riedelianum*).

When analyzing the centesimal composition of the starches, marked differences were observed between the two materials According to the statistical analysis, significant differences were found between most of the evaluated parameters, except for protein content. Turmeric starch had a lower moisture content (13.77%) compared to mangarito starch (29.68%). Martins et al. [4] and Ávila et al. [9] found lower moisture values, 10.57 ± 0.15 g 100 g^−1^ and 7.40 ± 0.37 g 100 g^−1^, when also evaluating starch isolated from *X. riedelianum* rhizomes. Moisture content can directly influence the microbiological stability and shelf life of the product. The lower moisture content of turmeric starch favors its conservation, making it more stable during storage.

Regarding the ash content, an indicator of the presence of minerals, turmeric starch presented 1.28%, significantly higher (*p* < 0.05) than that found in mangarito starch (0.15%). Maniglia et al. [3] found levels ranging from 4.4 ± 0.1 to 5.5 ± 0.1 g/100 g of starch obtained from the residue of turmeric dye extraction. This suggests a higher content of minerals or residual impurities in the turmeric sample, which may be related to the presence of bioactive compounds bound to the rhizome matrix, such as curcumin and inorganic salts.

The lipid content was also significantly higher (*p* < 0.05) in turmeric starch (5.26%) compared to mangarito starch (0.03%). These results are in agreement with the values reported by Ávila et al. [9] and Maniglia et al. [3] who observed levels of 0.06 ± 0.01 g/100 g and 5.0 ± 0.2 g/100 g of lipids for mangarito and turmeric starches, respectively. This discrepancy can be attributed to the presence of essential oils and lipophilic compounds naturally present in turmeric [3]. The high amount of lipids can negatively affect some technofunctional properties of starch, such as retrogradation and gelatinization capacity, although it can contribute positively in specific food formulations, as emulsifiers or in systems with controlled release of fat-soluble compounds.

No significant difference (*p* > 0.05) in protein content was observed between mangarito starch (2.24%) and turmeric starch (2.30%) samples. These findings are consistent with the data presented by Ávila et al. [9] and Maniglia et al. [3], who reported protein contents of 0.61 ± 0.17 g/100 g for mangarito starch and 3.0 ± 0.2 g/100 g for turmeric starch. The presence of proteins can interfere with the functionality of starch, such as film formation and paste viscosity, and is a relevant factor for industrial applications, especially in the food and biodegradable packaging industries.

The carbohydrate content, the main constituent of starch, was found to be significantly different (*p* < 0.05) between turmeric (77.39%) and mangarito (89.58%). Both values were lower than the starch content reported by Ávila et al. [9] for mangarito (91.81 ± 0.33 g/100 g). In general, the comparative analysis suggests that, while mangarito starch has a higher yield and lower content of interfering compounds (such as lipids), turmeric starch may stand out due to its lower moisture content and higher content of bioactive compounds, although at levels that may affect technological properties. The choice between one or the other will depend on the desired final application, whether in food formulations, biodegradable packaging or more specific industrial uses.

The amylose content of turmeric starch (55.13 ± 0.86%) was significantly higher (*p* < 0.05) than that of mangarito starch (25.91 ± 0.58%). Turmeric starches isolated under acidic (AS), aqueous (WS), and alkaline (KS) conditions exhibited apparent amylose contents of 42.8 ± 1.3, 51.5 ± 0.8, and 38.5 ± 1.1 g per 100 g of starch (dry basis), respectively [3]. For comparison, the amylose contents of starches from *Xanthosoma sagittifolium* and *Colocasia esculenta*, determined using the colorimetric method, were 35.34 ± 0.65% and 30.62 ± 0.16%, respectively [23]. In the study conducted by Wang et al. [10], kidney bean starch exhibited the highest amylose content (35.62%), followed by fern root starch (26.21%), potato starch (26.48%), and corn starch (23.99%). Cassava starch showed the lowest amylose content (17.65%).

These differences highlight the distinct molecular organization and granule structures of the evaluated starches, which can directly influence their physicochemical and functional properties.

High-amylose starches, such as that from turmeric, tend to exhibit greater crystallinity, higher gelatinization temperatures, and lower swelling power due to the stronger intermolecular associations of amylose chains. These characteristics often result in firmer gels, higher retrogradation tendency, and greater resistance to enzymatic hydrolysis, making such starches suitable for applications requiring structural stability, such as in biodegradable films or products with slow digestibility [24,25].

In contrast, the lower amylose content observed in mangarito starch suggests a predominance of amylopectin, which typically leads to higher swelling capacity, lower gelatinization temperature, and reduced retrogradation [26]. These features favor its use in food systems where high viscosity, smooth texture, and freeze–thaw stability are desired, such as in sauces, fillings, and frozen desserts.

The marked difference in amylose content between the two starches can be attributed to their botanical origin and genetic background. Although both are rhizomatous species, they belong to distinct botanical families—*Zingiberaceae* and *Araceae*—which differ in enzymatic activity and biosynthetic pathways related to starch synthesis. Consequently, the starch from turmeric behaves more like a high-amylose starch, whereas mangarito starch resembles typical root starches with higher amylopectin content.

### 3.3. Water Absorption Index and Solubility Index

Water solubility index (WSI) and water absorption index (WAI) are fundamental parameters in the technofunctional characterization of starches, as they provide information about their granular structure, interaction behavior and possible applications in food and industrial formulations [27]. These indices reflect the capacity of starch granules to absorb water, which results from the disruption of hydrogen bonds, the formation of new bonds between the hydroxyl groups of water and starch, and the consequent leaching and release of soluble components under specific temperature and agitation conditions [17,28].

The WSI of turmeric (*Curcuma longa*) starch was 2.36 ± 0.18%, whereas that of mangarito starch was 2.11 ± 0.18%, showing no statistically significant difference between samples (*p* > 0.05). A similar trend was observed for the WAI, with values of 2.88 ± 0.02 g/g for turmeric and 2.87 ± 0.04 g/g for mangarito (*p* = 0.676), indicating no significant variation between the two starches. According to Nuwamanya et al. [29], the swelling power at 80 °C was higher for cassava (8.58 g/g) and potato (8.44 g/g) starches compared with sweet potato (6.88 g/g). In contrast, cereal starches such as corn, wheat, millet, and sorghum exhibited lower values (5.17 g/g). In the same study, starch solubility was lower for potato (0.77 g/g) and sweet potato (0.58 g/g) than for cassava (1.23 g/g). Starch extracted from mango seed (*Mangifera indica*) showed a water absorption capacity of 0.16% [18].

In the present study, both starches exhibited similar functional behavior regarding solubility and water retention capacity, suggesting comparable granular organization and crystallinity. However, considering amylose content, turmeric starch showed significantly higher values (55.13 ± 0.86%) compared with mangarito starch (25.91 ± 0.58%; *p* < 0.05). Generally, a high amylose content tends to reduce solubility and water absorption due to its linear structure and strong hydrogen bonding, which confer greater rigidity and lower permeability to the granules [30]. Thus, the slightly higher WSI and WAI values observed for turmeric starch, although not statistically significant, may indicate that other structural factors—such as the presence of amorphous regions, lower granular compaction, or residual impurities (salts, sugars, non-starch polysaccharides, proteins, lipids, or curcumin) [31,32], which act by influencing the behavior of starch in aqueous medium, affected by competitive interactions of the starch with these constituents.

Conversely, the lower amylose content in mangarito starch is associated with a higher proportion of amylopectin, whose branched structure favors swelling and water absorption. According to Zhu [33], increased water absorption capacity is typically associated with higher amylopectin content, since its branched architecture provides a greater surface area and multiple interaction sites with water compared to amylose. However, the absence of significantly higher WSI or WAI values for mangarito starch suggests that its more intact and crystalline granule structure may limit water diffusion, offsetting the effect of its higher amylopectin content. Therefore, despite the contrasting amylose levels, the overall functional behavior (WSI and WAI) reflects a structural balance in which turmeric starch combines higher amylose with lower granular order, while mangarito starch combines lower amylose with greater crystalline compactness.

In summary, the correlation between amylose content and techno-functional indices indicates that, despite significant compositional differences, solubility and water absorption properties did not differ statistically. This finding demonstrates that, beyond amylose content, other structural attributes—such as crystallinity degree, granule size, and the presence of non-starch components—play a decisive role in determining the functional performance of these starches. Overall, both starches exhibited relatively high WAI values (>2.0 g/g), making them promising candidates for applications requiring good hydration and water retention capacity, such as processed meat products, thickening agents for sauces, and gluten-free formulations. The similarity in WSI and WAI values also suggests that turmeric and mangarito starches could be used interchangeably in food or industrial systems requiring controlled dispersion and gelatinization properties, with the choice depending mainly on raw material availability and cost considerations [34].

### 3.4. Viscoamylograph Properties

**Figure 1:**
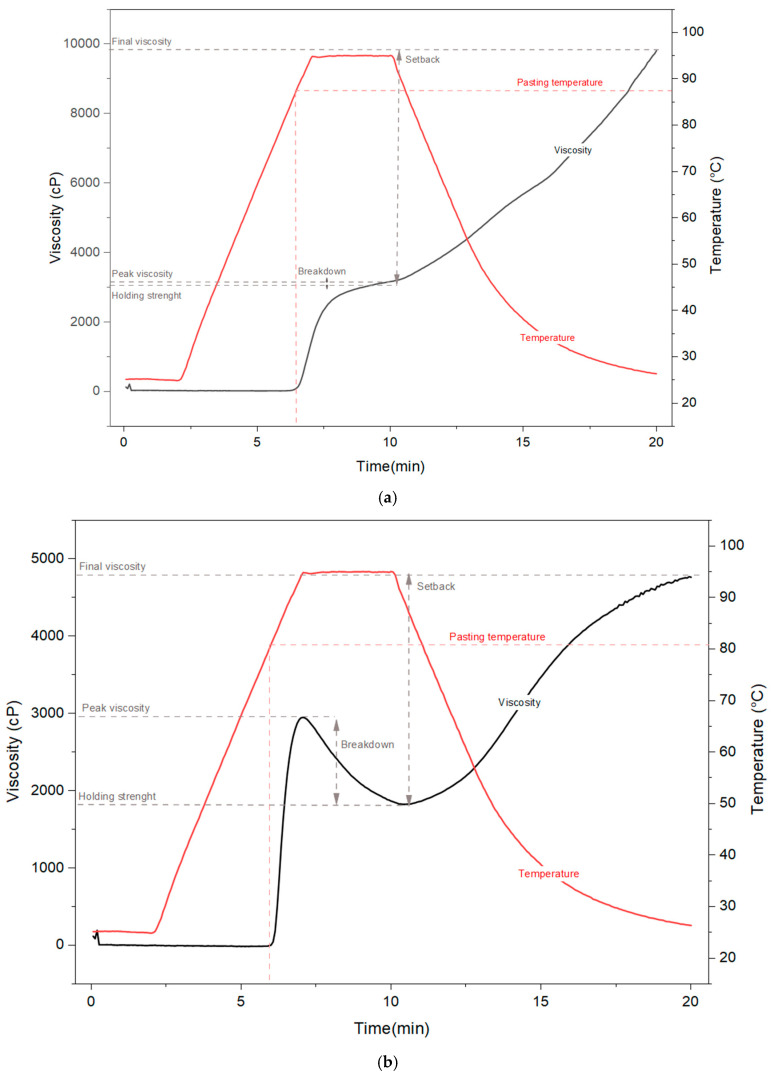
Viscoamylographic profile of (**a**) turmeric (*Curcuma longa* L.) and (**b**) mangarito (*Xanthosoma riedelianum*) starches.

Viscoamylographic analysis provides essential information on the behavior of starches during heating, temperature maintenance and cooling, and is widely used to characterize their functionality in food and industrial systems. Parameters such as pasting temperature, peak viscosity, viscosity breakdown, retrogradation and final viscosity directly reflect the molecular structure, degree of branching and integrity of starch granules [35,36] (Figure 1).

Regarding the pasting temperature, turmeric starch exhibited an initial gelatinization temperature of 84.7 °C, while mangarito starch began to gelatinize at 81.85 °C. This indicates that turmeric starch requires slightly higher thermal energy to initiate granule swelling, suggesting a more ordered or crystalline structure.

The peak viscosity represents the maximum expansion and swelling of the starch granules during heating [37]. In the viscoamylographic curve (Figure 1), the parameter Visc(cP)A corresponds to this peak point, recorded at 3147.5 cP for turmeric starch and 2948.5 cP for mangarito starch. The higher value for turmeric starch indicates a greater swelling capacity and the ability to form more viscous pastes during heating, which may be associated with its solubility and water absorption index.

In addition, the amount of amylose present in the starch has a significant influence on its gelatinization characteristics. However, if the starch granules suffer damage during processing, amylose and amylopectin may be released, contributing to an increase in paste viscosity due to enhanced water binding and molecular entanglement [38,39].

Viscosity breakdown results from the degradation of swollen granules during agitation and heating, reflecting the thermal stability of the paste [40]. This parameter is represented in Figure 1 by the difference between Visc(cP)A (peak) and Visc(cP)B (viscosity after breakdown). Turmeric starch showed a small viscosity drop (83.5 cP), maintaining a post-breakdown viscosity of 3064 cP, which indicates high thermal stability of the paste. Conversely, mangarito starch exhibited a sharp breakdown, decreasing from 2948.5 cP to 1124.5 cP (a reduction of 1824 cP), demonstrating that its granules are more susceptible to disintegration under heat and shear, and thus exhibit lower thermal stability.

During cooling stage, both starches demonstrated a tendency toward retrogradation, characterized by the reassociation of amylose chains via hydrogen bonds and partial recrystallization of the structure [37]. As shown in Figure 1, the mean viscosity (Visc(cP) mean) corresponds to the final equilibrium viscosity after the cooling stage, reaching 9806 cP for turmeric starch and 4763.5 cP for mangarito starch. This pronounced difference highlights the distinct pasting behavior of the two starches. In a broader context, Sánchez et al. [41] analyzed approximately 4050 cassava genotypes from a global collection cultivated in Colombia and reported substantial variability in their pasting properties. The values for peak viscosity (PV), breakdown (BD), setback (SB), and peak gelatinization temperature (PT) ranged from 146 to 1505 cP, 28.1 to 859 cP, −702 to 273 cP, and 58.8 to 71.2 °C, respectively, demonstrating the extensive functional diversity among cassava starches. Moreover, starches from other botanical sources also exhibit marked differences in their thermal behavior. Corn (*Zea mays*) starch shows a relatively wide gelatinization range, with onset temperatures (To) between 65.6 and 69.0 °C, peak temperatures (Tp) from 69.9 to 74.0 °C, and conclusion temperatures (Tc) between 75.1 and 79.7 °C [42]. In contrast, rice (*Oryza sativa*) starch displays narrower ranges, with To varying from 61.6 to 64.6 °C, Tp from 66.6 to 69.3 °C, and Tc from 72.1 to 73.9 °C, indicating a more uniform thermal transition process [43]. Taken together, these findings confirm that botanical origin plays a decisive role in determining the thermal and pasting properties of starches, resulting in distinct gelatinization profiles across different plant species.

The high final viscosity and the small difference between the peak and final viscosity in turmeric starch indicate a strong tendency toward retrogradation and formation of a rigid structure at room temperature. This property can be advantageous in products requiring firm gelation and high stability, such as gelled desserts, moldable doughs, or biodegradable films with structural integrity. In contrast, mangarito starch, with its lower final viscosity and reduced retrogradation (1824 cP), appears more suitable for applications demanding softer textures and lower recrystallization, such as sauces, creams, or formulations that must maintain a smooth consistency for extended periods.

### 3.5. Scanning Electron Microscopy (SEM)

**Figure 2:**
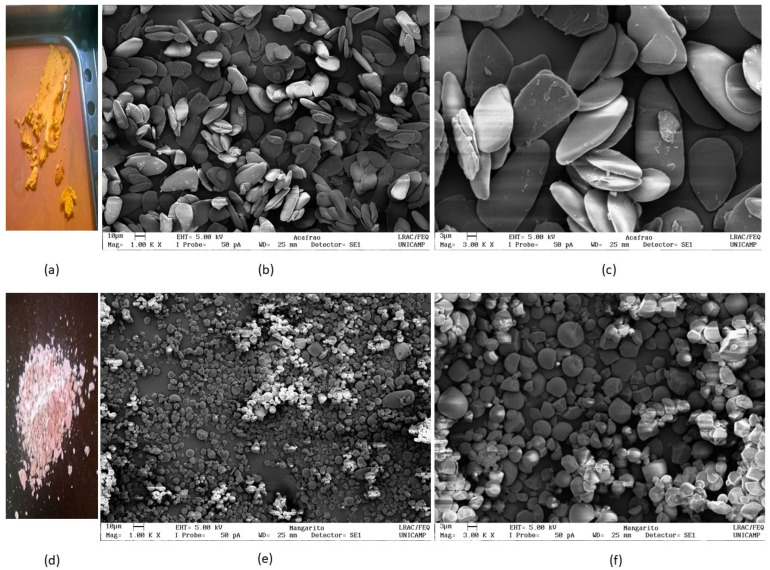
Photographic images and scanning electron microscopy (SEM) of turmeric (*Curcuma longa* L.) (**a**–**c**) and mangarito (*Xanthosoma riedelianum*) (**d**–**f**) starches. Magnification of 1 k ((**b**,**e**), bar 10 µm); 3 k ((**c**,**f**), bar 3 µm).

Figure 2a shows that turmeric starch exhibits an orange color, attributed to the presence of curcuminoids, phenolic compounds responsible for the pigmentation of the rhizome [3,5]. In contrast, mangarito starch displays a whitish color (Figure 2d), which is characteristic of pigment-free starches.

The micrographs (Figure 2b,c) indicate that turmeric starch granules present heterogeneous morphologies, including triangular, ellipsoidal, and oval shapes, often with a pointed end. The occurrence of ellipsoidal granules with dimensions between 20 and 50 μm has already been reported for this species [3]. This differentiated morphology contrasts with the more common forms found in traditional starches, such as corn [44] or potato [45], suggesting an influence of the anatomical structure of *Curcuma longa* L. rhizomes.

The granules of native tapioca starch and native waxy tapioca starch exhibited predominantly rounded to truncated morphologies, with an average diameter of approximately 12 μm, as observed under optical microscopy. In contrast, normal rice starch and waxy rice starch granules showed angular and polygonal shapes, with comparable average sizes of around 5 μm, as determined by optical microscopy analysis [46].

The morphological analysis of mangarito starch (Figure 2e,f) revealed well-defined granules, predominantly rounded, although some fractured granules were also observed. According to Pérez et al. [23], starches from *Xanthosoma sagittifolium* exhibit small rounded or large truncated ellipsoidal granules, with diameters ranging from 2 to 12.5 μm. For comparison, native and waxy tapioca starches show rounded to truncated morphologies, with an average diameter of approximately 12 μm, while normal and waxy rice starch granules exhibit angular and polygonal shapes with sizes close to 5 μm [46]. In mangarito starch, the combination of rounded and angular granules suggests a consistent granular organization, with potential regions of higher compaction.

Additionally, aggregated structures adjacent to the starch granules were observed in both turmeric and mangarito samples, in agreement with the findings reported by Maniglia et al. [3], for turmeric starch.

### 3.6. X-Ray Diffraction (XRD)

X-ray diffractometry (XRD) analysis allows the identification of the crystalline structure of starch granules, differentiating the different types of crystallinity A, B and C, which directly influence functional properties such as solubility, thermal resistance, gelatinization temperature and tendency to retrogradation [47]. The crystalline arrangement of starch is largely determined by the chain-length distribution of amylopectin, the degree of molecular packing inside the granules, and the amount of water associated with the structure [48]. Type A is characterized by major peaks at 15°, 17°, 18°, and 20° (2θ); type B exhibits peaks at 5°, 6°, 15°, 17°, 18°, and 23°; and type C exhibits a mixed pattern of types A and B, with peaks at 5.5°, 15°, 17°, 22°, and 23° [49,50]. Type C is most frequently observed in starches extracted from rhizomes [50]. A V-type polymorph may also arise when granules undergo swelling or form amylose–lipid complexes, generating additional diffraction features [48].

Examples in the literature include potato starch with a typical B-type pattern (reflections at ~17.2° and 21.7°), corn starch with an A-type profile (weak reflections at ~19.4° and 15.8° and an intense doublet around 17–17.4°), and cassava starch exhibiting a C-type pattern dominated by A-type peaks at ~15.3°, 23°, and an intense reflection near 17–17.6° [48].

**Figure 3:**
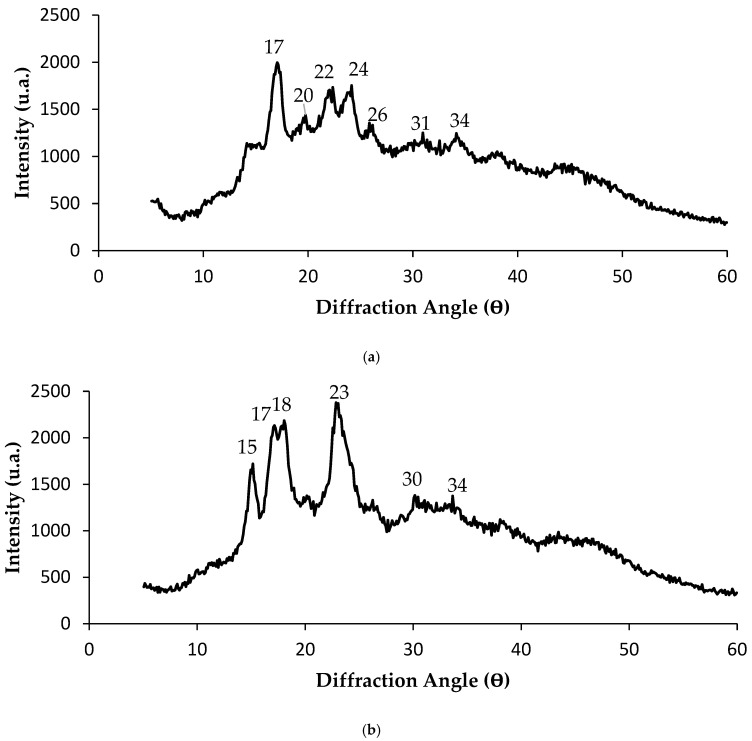
X-ray diffractometry (XRD) analysis results: (**a**) turmeric (*Curcuma longa* L.) and (**b**) mangarito (*Xanthosoma riedelianum*) starches.

Within this structural framework, the X-ray diffraction (XRD) pattern obtained for turmeric starch reveals the typical behavior of semicrystalline natural polymeric materials, characterized by a pronounced amorphous halo overlaid by broad and low-intensity peaks. The main reflections observed between 15° and 25° (2θ), particularly those around 17°, 20°, 22°, and 24°, indicate the presence of residual crystalline domains, although poorly defined (Figure 3a). These peaks are consistent with A-type or hybrid A/B-type structures, commonly associated with the helical arrangement of amylopectin. Secondary reflections at approximately 26°, 31°, and 34° may correspond to minor contributions from recrystallized amylose or trace minerals naturally present in the rhizome, reinforcing the heterogeneous structural nature of the extracted starch.

This qualitative interpretation is supported by Maniglia et al. [3] who identified significant peaks at 2θ ≈ 5.6°, 15°, 17°, 19.6°, and 22° in turmeric starches isolated in different media (acidic, aqueous, and alkaline), which are characteristic of B-type structures. Furthermore, peaks at 2θ ≈ 17° and 22° have been associated with lignocellulosic residues [4,19,51] while reflections at 15° and 17° may indicate the presence of curcuminoids [3,52]. These observations explain the coexistence of signals attributable both to the starch matrix and to bioactive and structural components of the rhizome, corroborating the hybrid and partially disordered nature of the material.

The predominance of a broad amorphous halo throughout the pattern suggests that turmeric starch possesses low structural order. This characteristic may be related not only to the presence of non-starchy compounds but also to the intrinsic arrangement of amylopectin. According to Costa et al. [53] longer branched chains with more widely spaced branch points favor the formation of the B-type polymorph, which is associated with a more open structural arrangement, higher water retention capacity, and a stronger tendency to form firm gels. These structural traits justify the functional behavior observed experimentally, including the high final viscosity (9806 cP) and the pronounced retrogradation detected in the viscoamylographic assays. The molecular reassociation during cooling, facilitated by long amylopectin chains and the availability of water, explains the increase in viscosity and the marked retrogradation.

Overall, the reduced semicrystalline structure of turmeric starch promotes higher solubility, easier gelatinization, and enhanced interaction with plasticizers—features that are advantageous for the development of biodegradable films and polymeric blends. However, properties such as thermal stability and mechanical resistance may be limited by the low structural organization, highlighting the need for modification strategies such as crosslinking, incorporation of nanoparticles, or blending with more structured polymers to adjust the final performance of the material according to the intended application.

In contrast, the X-ray diffractogram (XRD) of mangarito starch presents the characteristic profile of a semicrystalline polysaccharide, marked by the presence of a broad amorphous halo superimposed on a restricted set of distinct peaks. The main reflections are located at approximately 2θ ≈ 15°, 18°, 23°, 30°, and 34°, each contributing to the understanding of the supramolecular organization of the starch granules (Figure 3b).

The peaks observed at 15° and 18° are commonly associated with the type A crystalline pattern, typical of cereal starches [47], and indicate the presence of short-range order in the amylopectin double helices. The most intense peak near 23° suggests a slightly higher level of crystallinity, indicating more compact regions within the amylopectin clusters or the formation of V-type complexes. The broader reflections at 30° and 34° may correspond to weakly ordered domains or inorganic residues naturally present in mangarito rhizomes, reinforcing the partially organized character of the structure.

This structural configuration is consistent with the literature’s understanding of the influence of amylopectin organization on the formation of crystalline types. According to Costa et al. [53], shorter branched chains with closer branching points favor the formation of the type A crystalline polymorph. This arrangement, typical of cereal starches such as wheat and rice [47], is associated with a more compact molecular packing, resulting in lower water absorption and greater thermal resistance.

The structural results are directly corroborated by the technofunctional behavior of the material. Mangarito starch showed greater stability to retrogradation and lower final viscosity (4763.5 cP), reflecting a lower propensity for molecular reorganization during cooling. This characteristic is aligned with type A, whose more compact structure limits the rearrangement of chains after gelatinization. As a result, this starch shows potential for applications requiring more fluid textures, greater thermal stability, and lower syneresis, such as sauces, creamy desserts, and colloidal systems.

In addition, XRD reveals that mangarito starch has a supramolecular organization consistent with a predominantly A-type material, with moderately defined crystalline regions, on an essentially amorphous matrix. This structure explains both its functional behavior and its potential application in biodegradable polymeric systems that demand good processability, stability, and low retrogradation tendency.

### 3.7. Differential Scanning Calorimetry (DSC)

Differential scanning calorimetry (DSC) is widely used in characterizing the thermal stability of biopolymeric materials. The method consists of measuring the difference in heat flow between the sample and a reference substance as a function of temperature or time, under controlled heating [54]. DSC curves provide relevant thermal parameters, such as the glass transition temperature range (Tg), which indicates the mobilization of the amorphous polymer chains during heating [12].

**Figure 4:**
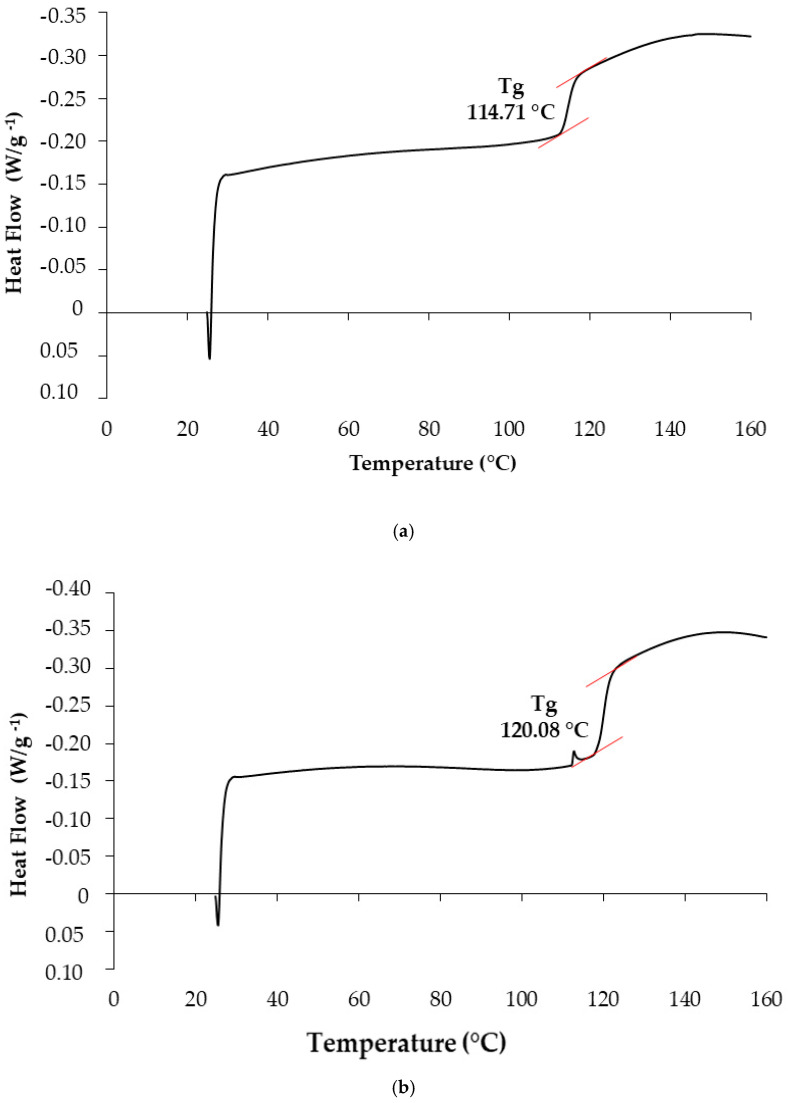
Differential Scanning Calorimetry (DSC) analysis results: (**a**) turmeric (*Curcuma longa* L.) and (**b**) mangarito (*Xanthosoma riedelianum*) starches.

The thermogram of turmeric starch shown in Figure 4a indicated a glass transition temperature (Tg) of 114.71 °C, with the transition beginning at approximately 113.18 °C and ending near 114.74 °C. For mangarito starch, the observed Tg was 120.08 °C, starting around 118.56 °C and ending at approximately 120.12 °C (Figure 4b). In the study conducted by Chuang et al. [55], the glass transition temperature (Tg) of potato starch decreased from 161.72 °C to 141.91 °C as the moisture content increased from 3.7% (*w*/*w*; RH 11%) to 18.8% (*w*/*w*; RH 75%). Similarly, Chang et al. [56] found that increasing the moisture content from 4.5% to 26% led to a marked decrease in the glass transition temperature of cassava starch, from 175 °C to 34 °C.

The glass transition temperature (Tg) of starches is strongly influenced by moisture content, the relative proportions of amylose and amylopectin, and the intermolecular interactions between the polymer chains and low molecular weight cosolutes [57,58,59]. Water, acting as a plasticizing agent, increases the segmental mobility of amorphous regions, resulting in a reduction in Tg [55]. Furthermore, the molecular arrangement of amylose and amylopectin chains, associated with hydrogen bond density and the degree of crystallinity, also exerts a significant effect on the thermal behavior of the material.

The observed differences in Tg between turmeric starch and mangarito starch can be attributed to variations in their chemical composition (Table 1). Turmeric starch showed a higher moisture and lipid content compared to mangarito starch. These constituents can act as plasticizing agents, reducing intermolecular interactions between polymer chains and, consequently, decreasing Tg [55]. Furthermore, turmeric starch showed a higher amylose content (55.13%) and the possible presence of bioactive compounds, such as curcuminoids, which can also interfere with the molecular organization of the starch matrix, promoting lower structural rigidity [60].

On the other hand, mangarito starch, with a higher total carbohydrate content and lower lipid and moisture content, showed a higher Tg, indicating greater thermal stability and lower molecular mobility. This characteristic may be associated with the more compact structure of the amorphous regions and the lower presence of plasticizing components, which confers greater thermal resistance to the material.

A high glass transition temperature (Tg) is a desirable property for polymers intended for film production. At temperatures below the Tg—such as those typically used during the drying process—the available thermal energy is insufficient to induce significant conformational changes in the polymer chains. Consequently, after solvent evaporation during film formation, the polymer tends to remain in the glassy state, exhibiting a rigid and stable structure that contributes to the development of films with enhanced mechanical strength [61].

### 3.8. Thermogravimetric Analysis (TGA)

Thermogravimetric analysis was performed to evaluate the thermal stability and degradation profile of turmeric (*Curcuma longa* L.) and mangarito (*Xanthosoma riedelianum*) starches. Although the thermal decomposition curves of both starches show similar patterns, variations were observed in the initial temperatures, decomposition peaks, and mass loss rates in each phase.

**Figure 5:**
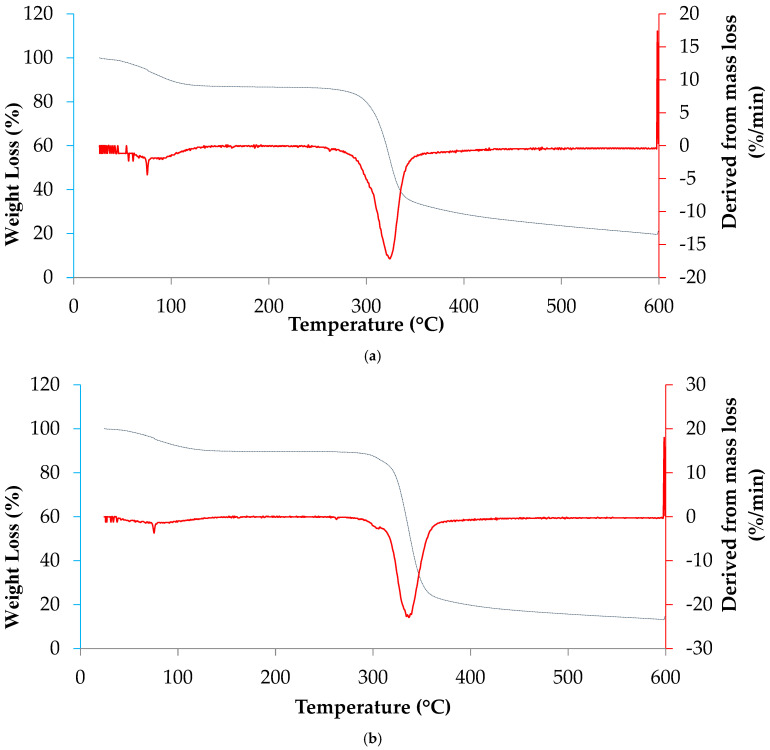
Mass loss profiles (blue line, %) and derivative mass loss rate (red line, %/min), obtained by thermogravimetric analysis (TGA), for: (**a**) turmeric (*Curcuma longa* L.) and (**b**) mangarito (*Xanthosoma riedelianum*) starches.

Turmeric starch displayed three major stages of mass loss (Figure 5a). The first, occurring between approximately 30–120 °C, corresponded to the evaporation of surface and weakly bound water [62], accounting for a modest initial mass decrease. The second, and most significant, event occurred between 250 and 330 °C, with the maximum rate of decomposition at ~300 °C, attributable to the breakdown of amylose and amylopectin chains and to the collapse of semicrystalline domains [63]. Decomposition proceeded over a relatively broad temperature range, indicating a less compact granular organization and a predominance of amorphous regions, which is consistent with its low crystallinity (~19%). For starch derived from cassava peel, cassava bagasse, and common starch, the initial decomposition temperatures were approximately 295.6 °C, 305.1 °C, and 294.5 °C, respectively [62]. Above 330 °C, a slow carbonization stage led to minimal residual mass at 600 °C [12]. This behavior is characteristic of B-type starches, which generally present higher water content, more open crystalline arrangements, and lower thermal stability compared with A-type counterparts [47,53].

Mangarito starch exhibited a distinct thermal profile, indicating a structurally more stable and compact material. The initial mass loss (30–120 °C) was lower than that of turmeric starch, reflecting reduced water adsorption (Table 1). The main degradation stage occurred between 270 and 340 °C, with a sharp DTG peak at approximately 300–340 °C (Figure 5b), corresponding to the rapid decomposition of amylose/amylopectin chains and collapse of well-organized crystalline domains. Degradation occurred more abruptly and within a narrower temperature range compared with turmeric starch, consistent with the presence of a predominantly A-type crystalline pattern [47,53]. Above 340 °C, a gradual carbonization process continued up to 600 °C, resulting in a greater residual mass, further confirming its superior thermal resistance [12].

The thermal behavior of the two starches clearly reflects their distinct molecular architectures. Turmeric starch, which exhibits B-type crystallinity and a more open supramolecular arrangement, undergoes progressive degradation over a broader temperature range and demonstrates lower thermal stability. In contrast, mangarito starch, characterized by a compact A-type crystalline structure, shows a more concentrated and intense main degradation event and higher thermal resistance. These differences are strongly aligned with their techno-functional properties: the higher amorphous fraction of turmeric starch explains its higher final viscosity and more pronounced retrogradation tendency, whereas the compact arrangement of mangarito starch contributes to its lower final viscosity, reduced retrogradation, and enhanced thermal stability—attributes desirable in applications requiring fluid texture, thermal robustness, and resistance to syneresis, such as sauces, colloidal systems, and creamy desserts.

## 4. Conclusions

This study highlights the distinct physicochemical, morphological, and thermal characteristics of starches extracted from turmeric (*Curcuma longa* L.) and mangarito (*Xanthosoma riedelianum*). Mangarito starch exhibited a higher extraction yield (11.6%) compared to turmeric starch (5.6%), indicating its potential as a more efficient source of starch for industrial applications. Proximate analysis revealed higher amylose content in turmeric starch and greater total carbohydrate content and lower moisture in mangarito starch, reflecting functional differences relevant for processing and formulation.

Rheological and thermal analyses demonstrated that mangarito starch possesses superior structural stability and thermal resistance, associated with a predominantly A-type crystalline pattern, whereas turmeric starch, characterized by a higher proportion of amorphous regions and lower crystallinity, displayed lower thermal stability. Morphological and X-ray diffraction analyses corroborated these findings, with mangarito granules showing a uniform and well-defined structure compared to the heterogeneous granules of turmeric starch. Turmeric starch showed higher viscosity and setback values than mangarito starch, indicating a greater retrogradation tendency.

These findings have significant implications for the food and biopolymer industries. Mangarito starch is particularly suitable for applications requiring high thermal and structural stability, while turmeric starch could be employed in formulations where high amylose content is desirable.

Future research should focus on the physicochemical modification of these starches to optimize functional properties and explore their application in biodegradable materials, food matrices, and pharmaceutical systems. The comprehensive characterization provided herein establishes a foundation for the valorization of unconventional starch sources in industrial applications.

## Figures and Tables

**Table 1:** Proximate composition of turmeric (*Curcuma longa* L.) and mangarito (*Xanthosoma riedelianum*) starches.

Proximate Composition	Turmeric Starch (%)	Mangarito Starch (%)
Moisture	13.77 ± 0.00 a	7.91 ± 0.11 b
Ash	1.28 ± 0.00 a	0.15 ± 0.06 b
Lipids	5.26 ± 0.01 a	0.03 ± 0.03 b
Proteins	2.30 ± 0.00 a	2.24 ± 0.02 a
Total carbohydrates	77.39 ± 0.81 b	89.58 ± 0.13 a
Amylose content	55.13 ± 0.86 a	25.91 ± 0.58 b

Same letters in the same row show no statistical difference (*p* > 0.05).

## Data Availability

The original contributions presented in this study are included in the article. Further inquiries can be directed to the corresponding author.

## References

[1] Carvalho H.J.M., Barcia M.T., Schmiele M. (2024). Non-Conventional Starches: Properties and Potential Applications in Food and Non-Food Products. Macromol.

[2] Shoukat R., Cappai M., Pilia L., Pia G. (2025). Rice Starch Chemistry, Functional Properties, and Industrial Applications: A Review. Polymers.

[3] Maniglia B.C., Silveira T.M.G., Tapia-Blácido D.R. (2022). Starch isolation from turmeric dye extraction residue and its application in active film production. Int. J. Biol. Macromol..

[4] Martins M.M.M., Souza D.C.D., Botrel N., Resende L.V., Pereira J. (2020). *Xanthosoma riedelianum* starch for use in the food industry. Pesqui. Agropecu. Bras..

[5] Aguilar G.J., Ferreira Da Silva Y., Augusto P.E.D., Perré P., Tapia-Blácido D.R. (2025). Morphological, structural, and functional properties of biobased cassava starch foam trays added with turmeric pigment extraction residue. J. Clean. Prod..

[6] Nguyen L., Govindasamy R., Mentreddy S.R. (2024). Turmeric trends: Analyzing consumer preferences and willingness to pay. Front. Sustain. Food Syst..

[7] Leonel M., Sarmento S.B.S., Cereda M.P. (2003). New starches for the food industry: Curcuma longa and Curcuma zedoaria. Carbohydr. Polym..

[8] Cereda M.P., Franco C.M.L., Daiuto E.R., Demiate I.M., Carvalho L.J.C.B., Leonel M., Vilpoux O.F., Sarmento S.B.S. (2001). Propriedades Gerais do Amido.

[9] De Ávila R., Ascheri D.P.R., Ascheri J.L.R. (2012). Caracterização dos Rizomas Filhos e da Fécula do Mangarito (*Xanthosoma mafaffa* Schott) e Elaboração de Filmes Biodegradáveis. Bol. CEPPA.

[10] Wang X., Reddy C.K., Xu B. (2018). A systematic comparative study on morphological, crystallinity, pasting, thermal and functional characteristics of starches resources utilized in China. Food Chem..

[11] Cruz R., El Dash A. (1984). Amido de chuchu (Seichium edule, Swartz). Efeito de fosfatação em sua viscosidade. Bol. SBCTA.

[12] Nogueira G.F., Fakhouri F.M., de Oliveira R.A. (2018). Extraction and characterization of arrowroot (*Maranta arundinaceae* L.) starch and its application in edible films. Carbohydr. Polym..

[13] Baur F.J., Ensminger L.G. (1977). The association of official analytical chemists (AOAC). J. Am. Oil Chem. Soc..

[14] Zavareze E.d.R., Halal S., Pereira J., Radünz A., Elias M., Dias A. (2009). Caracterização química e rendimento de extração de amido de arroz com diferentes teores de amilose. Braz. J. Food Technol..

[15] Schoch T.J. (1964). (28) Swelling Power and solubility of Granular Starches. Methods in Carbohydrate Chemistry.

[16] American Association of Cereal Chemists (2000). Approved Methods Committee, Approved Methods of the American Association of Cereal Chemists.

[17] Souza D.C.D., Silva R.D.J., Guerra T.S., Silva L.F.L.E., Resende L.V., Pereira J. (2019). Characterization of arrowroot starch in different agronomic managements. Rev. Ceres.

[18] Akhter M.J., Sarkar S., Rayhanujjaman M., Kabir M.S., Hosain M.M. (2024). Characterization of mango seed kernel starch: Extraction and Analysis. Food Chem. Adv..

[19] Choudhary P., Devi T.B., Dawange S.P., Narsaiah K. (2023). Valorization of Mango By-Products: Extraction and Characterization of Starch from Seed Kernels. Starch Stärke.

[20] Panchal P.M., Singh B.K. (2024). Effect of Physical and Chemical Extraction Methods on Yield of Chayote (*Sechium edule*) Tuber Starch. J. Sci. Res. Rep..

[21] Dorantes-Fuertes M.-G., López-Méndez M.C., Martínez-Castellanos G., Meléndez-Armenta R.Á., Jiménez-Martínez H.-E. (2024). Starch Extraction Methods in Tubers and Roots: A Systematic Review. Agronomy.

[22] Zou J., Xu M., Wen L., Yang B. (2020). Structure and physicochemical properties of native starch and resistant starch in Chinese yam (*Dioscorea opposita* Thunb.). Carbohydr. Polym..

[23] Pérez E., Schultz F.S., De Delahaye E.P. (2005). Characterization of some properties of starches isolated from *Xanthosoma sagittifolium* (tannia) and *Colocassia esculenta* (taro). Carbohydr. Polym..

[24] Zhong Y., Liu L., Qu J., Blennow A., Hansen A.R., Wu Y., Guo D., Liu X. (2020). Amylose content and specific fine structures affect lamellar structure and digestibility of maize starches. Food Hydrocoll..

[25] Zhong Y., Tai L., Blennow A., Ding L., Herburger K., Qu J., Xin A., Guo D., Hebelstrup K.H., Liu X. (2023). High-amylose starch: Structure, functionality and applications. Crit. Rev. Food Sci. Nutr..

[26] Cornejo-Ramírez Y.I., Martínez-Cruz O., Del Toro-Sánchez C.L., Wong-Corral F.J., Borboa-Flores J., Cinco-Moroyoqui F.J. (2018). The structural characteristics of starches and their functional properties. CyTA—J. Food.

[27] Friero I., Martínez-Subirà M., Romero M.-P., Moralejo M. (2024). Improving functional and nutritional profiles of barley flours with diverse starch types through pearling. Food Chem..

[28] Wani I.A., Sogi D.S., Gill B.S. (2012). Physicochemical properties of acetylated starches from some Indian kidney bean (*Phaseolus vulgaris* L.) cultivars. Int. J. Food Sci. Technol..

[29] Nuwamanya E., Baguma Y., Wembabazi E., Rubaihayo P. (2011). A Comparative study of the physicochemical properties of starches from root, tuber and cereal crops. Afr. J. Biotechnol..

[30] Biduski B., Silva W.M.F.D., Colussi R., Halal S.L.D.M.E., Lim L.-T., Dias Á.R.G., Zavareze E.D.R. (2018). Starch hydrogels: The influence of the amylose content and gelatinization method. Int. J. Biol. Macromol..

[31] Scott G., Awika J.M. (2023). Effect of protein–starch interactions on starch retrogradation and implications for food product quality. Compr. Rev. Food Sci. Food Saf..

[32] Donmez D., Pinho L., Patel B., Desam P., Campanella O.H. (2021). Characterization of starch–water interactions and their effects on two key functional properties: Starch gelatinization and retrogradation. Curr. Opin. Food Sci..

[33] Zhu F. (2018). Relationships between amylopectin internal molecular structure and physicochemical properties of starch. Trends Food Sci. Technol..

[34] Wang Y., Ou X., Al-Maqtari Q.A., He H.-J., Othman N. (2024). Evaluation of amylose content: Structural and functional properties, analytical techniques, and future prospects. Food Chem. X.

[35] Won C., Jin Y.I., Chang D.-C., Kim M., Lee Y., Ganesan P., Lee Y.-K., Chang Y.H. (2017). Rheological, pasting, thermal and retrogradation properties of octenyl succinic anhydride modified potato starch. Food Sci. Technol..

[36] Schirmer M., Höchstötter A., Jekle M., Arendt E., Becker T. (2013). Physicochemical and morphological characterization of different starches with variable amylose/amylopectin ratio. Food Hydrocoll..

[37] Wang Y., Chen L., Yang T., Ma Y., McClements D.J., Ren F., Tian Y., Jin Z. (2021). A review of structural transformations and properties changes in starch during thermal processing of foods. Food Hydrocoll..

[38] Huang J., Zhao L., Man J., Wang J., Zhou W., Huai H., Wei C. (2015). Comparison of physicochemical properties of B-type nontraditional starches from different sources. Int. J. Biol. Macromol..

[39] Cai J., Cai C., Man J., Zhou W., Wei C. (2014). Structural and functional properties of C-type starches. Carbohydr. Polym..

[40] Li W., Shan Y., Xiao X., Luo Q., Zheng J., Ouyang S., Zhang G. (2013). Physicochemical Properties of A- and B-Starch Granules Isolated from Hard Red and Soft Red Winter Wheat. J. Agric. Food Chem..

[41] Sánchez T., Salcedo E., Ceballos H., Dufour D., Mafla G., Morante N., Calle F., Pérez J.C., Debouck D., Jaramillo G. (2009). Screening of Starch Quality Traits in Cassava (*Manihot esculenta* Crantz). Starch Stärke.

[42] Sandhu K., Singh N. (2007). Some properties of corn starches II: Physicochemical, gelatinization, retrogradation, pasting and gel textural properties. Food Chem..

[43] Rodríguez-Torres D., Murillo-Arango W., Vaquiro-Herrera H.A., Solanilla-Duque J.F. (2017). Thermal and physicochemical properties of starches from three Colombian rice varieties. Agron. Colomb..

[44] Liu Z., Zhao Y., Zheng J., Wang Z., Yan X., Zhang T. (2024). Influence of enzymatic extraction on the properties of corn starch. Food Biosci..

[45] Bodor K., Tamási B., Len A., Keresztesi Á., Szép R., Bodor Z. (2025). Investigation of the properties of different potato varieties and analysis of the starch structure. Appl. Food Res..

[46] Boonkor P., Sagis L.M.C., Lumdubwong N. (2022). Pasting and Rheological Properties of Starch Paste/Gels in a Sugar-Acid System. Foods.

[47] Zhang B., Li X., Liu J., Xie F., Chen L. (2013). Supramolecular structure of A- and B-type granules of wheat starch. Food Hydrocoll..

[48] Teixeira B.S., Garcia R.H.L., Takinami P.Y.I., Del Mastro N.L. (2018). Comparison of gamma radiation effects on natural corn and potato starches and modified cassava starch. Radiat. Phys. Chem..

[49] Huang H., Jiang Q., Chen Y., Li X., Mao X., Chen X., Huang L., Gao W. (2016). Preparation, physico–chemical characterization and biological activities of two modified starches from yam (*Dioscorea opposita* Thunb.). Food Hydrocoll..

[50] Hornung P.S., Granza A.G., de Oliveira C.S., Lazzarotto M., Schnitzler E. (2015). Study of the Effects of Ultraviolet Light and Sodium Hypochlorite Solutions on Properties of Cassava Starch Granules. Food Biophys..

[51] Silva E.K., Martelli-Tosi M., Vardanega R., Nogueira G.C., Zabot G.L., Meireles M.A.A. (2018). Technological characterization of biomass obtained from the turmeric and annatto processing by using green technologies. J. Clean. Prod..

[52] Pandey K.U., Dalvi S.V. (2019). Understanding stability relationships among three curcumin polymorphs. Adv. Powder Technol..

[53] Costa M.S., Volanti D.P., Grossmann M.V.E., Franco C.M.L. (2018). Structural, thermal, and morphological characteristics of cassava amylodextrins. J. Sci. Food Agric..

[54] Zuo C., Zhang C. (2025). Standardizing differential scanning calorimetry (DSC) thermal decomposition temperatures at various heating rates of an energetic material as a threshold one. Energetic Mater. Front..

[55] Chuang L., Panyoyai N., Katopo L., Shanks R., Kasapis S. (2016). Calcium chloride effects on the glass transition of condensed systems of potato starch. Food Chem..

[56] Chang Y.P., Cheah P.B., Seow C.C. (2000). Plasticizing—Antiplasticizing Effects of Water on Physical Properties of Tapioca Starch Films in the Glassy State. J. Food Sci..

[57] Kasapis S. (2005). Glass Transition Phenomena in Dehydrated Model Systems and Foods: A Review. Drying Technol..

[58] Perdomo J., Cova A., Sandoval A.J., García L., Laredo E., Müller A.J. (2009). Glass transition temperatures and water sorption isotherms of cassava starch. Carbohydr. Polym..

[59] Renzetti S., Henket J., Raaijmakers E., Van Den Hoek I., Van Der Sman R. (2025). Hydrogen bond density and glass-transition temperature govern gelatinization and gel rheology in cereal and tuber starches. Curr. Res. Food Sci..

[60] Guo L., Liang Q., Du X. (2011). Effects of molecular characteristics of tea polysaccharide in green tea on glass transitions of potato amylose, amylopectin and their mixtures. Food Hydrocoll..

[61] Sarantópoulos C., Oliveira L.D., Padula M., Coltro L., Alves R.M., Garcia E.E. (2002). Embalagens plásticas flexíveis: Principais polímeros e avaliação de propriedades. Camp. CETEA/ITAL.

[62] Weligama Thuppahige V.T., Moghaddam L., Welsh Z.G., Wang T., Xiao H.-W., Karim A. (2023). Extraction and characterisation of starch from cassava (*Manihot esculenta*) agro-industrial wastes. LWT.

[63] Herniou-Julien C., Mendieta J.R., Gutiérrez T.J. (2019). Characterization of biodegradable/non-compostable films made from cellulose acetate/corn starch blends processed under reactive extrusion conditions. Food Hydrocoll..

